# Supplementary material for: Content-rich biological network constructed by mining PubMed abstracts
Source: BMC Bioinformatics. 2004 Oct 8;5:147. doi: 10.1186/1471-2105-5-147 (PMC528731; doi:10.1186/1471-2105-5-147)
Supplement: Additional File 2 — The original results of the above study (non-essential files are deleted to keep the file size under the limit set by BMC bioinformatics). [file 1471-2105-5-147-S2.bz2 › chilibotAdditionalFile2/dip05/679ID9514722E11088/html/MAUB_MAUA.html]

 


 **MAUB** and **MAUA** 
  
Found 74 abstracts in PubMed, retrieved 05.  
 

 What does Google say? 
 PDF only 
| .edu only 

---

**Interactive relationship** (e.g. stimulation, inhibition, etc)

**Inhibitory relationship**- alphaF55A MADH  [ **MAUA** ]   [ **MAUB** ]  exhibits an increased K d for cation dependent spectral changes and a decreased K d for cation dependent stimulation of the rate of gated ET from N quinol MADH  [ **MAUA** ]   [ **MAUB** ]  to amicyanin.  Ref: 12686138 Biochim Biophys Acta, 2003
**Neutral relationship**- For each mutant, the majority of the protein that was isolated was inactive and exhibited weaker subunit subunit interactions than native MADH  [ **MAUA** ]   [ **MAUB** ] .  Ref: 12641453 Biochemistry, 2003
- The biogenesis of the quinone cofactor tryptophan tryptophylquinone TTQ in methylamine dehydrogenase MADH  [ **MAUA** ]   [ **MAUB** ]  involves the post translational modification of two constitutive Trp residues Trp beta 57 and Trp beta 108 in Paracoccus denitrificans MADH  [ **MAUA** ]   [ **MAUB** ] .  Ref: 12641453 Biochemistry, 2003

**Non-interactive relationship** (e.g. studied together, co-existance, homology, etc.)

- Methylamine dehydrogenase MADH  [ **MAUA** ]   [ **MAUB** ]  possesses an alpha 2 beta 2 subunit structure with each smaller beta subunit possessing a tryptophan tryptophylquinone TTQ prosthetic group.  Ref: 12686138 Biochim Biophys Acta, 2003
- One of these, mauG, exhibits sequence similarity to diheme cytochrome c peroxidases and is required for the synthesis of the tryptophan tryptophylquinone TTQ prosthetic group of MADH  [ **MAUA** ]   [ **MAUB** ] .  Ref: 12809487 Biochemistry, 2003
- The biosynthesis of methylamine dehydrogenase MADH  [ **MAUA** ]   [ **MAUB** ]  from Paracoccus denitrificans requires four genes in addition to those that encode the two structural protein subunits.  Ref: 12809487 Biochemistry, 2003
- A stable binary MADH  [ **MAUA** ]   [ **MAUB** ]  amicyanin complex can be formed, and its crystal structure has been solved to 2.5 A resolution by Chen et al.  Ref: 12686162 Biochim Biophys Acta, 2003
- Methylamine dehydrogenase MADH  [ **MAUA** ]   [ **MAUB** ]  is produced by a range of gram negative methylotrophic and autotrophic bacteria, and allows the organisms to utilise methylamine as the sole source of carbon.  Ref: 12686162 Biochim Biophys Acta, 2003
- Site directed mutagenesis of either alphaPhe 55 or betaIle 107 a residue in the beta subunit which interacts with alphaPhe 55 converts MADH  [ **MAUA** ]   [ **MAUB** ]  into enzymes with specificities for long chain amines, amylamine or propylamine.  Ref: 12686138 Biochim Biophys Acta, 2003
- Site directed mutagenesis studies have revealed several roles for this residue in catalysis and electron transfer ET by MADH  [ **MAUA** ]   [ **MAUB** ] .  Ref: 12686138 Biochim Biophys Acta, 2003
- Paracoccus denitrificans produces two primary enzymes for the amine oxidation, tryptophan tryptophylquinone TTQ containing methylamine dehydrogenase MADH  [ **MAUA** ]   [ **MAUB** ]  and quinohemoprotein amine dehydrogenase QH AmDH.  Ref: 12686147 Biochim Biophys Acta, 2003
- These properties are in marked contrast with those of MADH  [ **MAUA** ]   [ **MAUB** ] , which shows high specificity to amicyanin.  Ref: 12686147 Biochim Biophys Acta, 2003
- To complete the catalytic cycle, MADH  [ **MAUA** ]   [ **MAUB** ]  is reoxidised via an electron transfer ET chain.  Ref: 12686162 Biochim Biophys Acta, 2003
